# Integrin β3 Orchestrates Hepatic Steatosis via a Novel CD36‐Dependent Lipid Uptake Complex

**DOI:** 10.1002/advs.202517455

**Published:** 2025-12-08

**Authors:** Ying Zhang, Lei Dai, Zhongquan Cui, Ye Zhou, Wenyuan Dong, Hongcheng Jiang, Mengwen Wang, Xiaodan Zhong, Wei Dong, Zhang Yue, Thati Madhusudhan, Hongjie Wang, Xiong Zhong Ruan, Hesong Zeng

**Affiliations:** ^1^ Department of Cardiology Tongji Hospital Tongji Medical College Huazhong University of Science and Technology Wuhan Hubei 430030 China; ^2^ Hubei Provincial Engineering Research Center of Vascular Interventional Therapy Wuhan Hubei 430030 China; ^3^ Hepatic Surgery Center Tongji Hospital Tongji Medical College Huazhong University of Science and Technology Wuhan Hubei 430030 China; ^4^ Department of Cardiovascular Surgery Union Hospital Tongji Medical College Huazhong University of Science and Technology Wuhan 430022 China; ^5^ Center for Thrombosis and Hemostasis University Medical Center Mainz Langenbeckstr. 1 55131 Mainz Germany; ^6^ Department of Cardiology Tongji Xianning Hospital Xianning Hubei 437011 China; ^7^ Centre for Lipid Research & Key Laboratory of Molecular Biology for Infectious Diseases (Ministry of Education) Institute for Viral Hepatitis Department of Infectious Diseases the Second Affiliated Hospital Chongqing Medical University Chongqing 400016 China; ^8^ John Moorhead Research Laboratory Centre for Nephrology University College London Medical School Royal Free Campus University College London London NW3 2PF UK

**Keywords:** integrin β3, MASH, metabolic stress, lipid uptake, CD36

## Abstract

In metabolic dysfunction‐related steatohepatitis (MASH), ITGB3 promotes hepatic fibrosis via activating hepatic stellate cells, but whether it directly regulates hepatic lipid metabolism through membrane‐scaffolding function and the underlying mechanisms remain unclear. Transcriptomic analyses of human and murine models of MASH revealed consistent upregulation of ITGB3 in hepatocytes. In mice, the hepatocyte‐specific overexpression of ITGB3 exacerbates diet‐induced obesity, insulin resistance, steatosis, and fibrosis, the deletion of ITGB3 alleviates these phenotypes. Additionally, the overexpression of DHHC5 reversed the hallmarks of MASH in ITGB3‐deficient mice, confirming the central role of DHHC5 in this process. Mechanistically, ITGB3 is a novel “accelerator” that directly increases CD36‐mediated fatty acid uptake by recruiting LYN, then modulating LYN protein stability, and triggering LYN proteasomal degradation. This degradation relieves LYN–mediated inhibition of DHHC5 and promotes ITGB3/DHHC5/CD36 complex formation, thereby enhancing DHHC5‐dependent CD36 palmitoylation and subsequent CD36‐mediated fatty acid uptake. Pharmacologic inhibition of ITGB3 using cyclic‐RGDfk peptide improved serum lipid profiles and hepatic steatosis. This study uncovers a previously unrecognized mechanism by which ITGB3 acts as a driver of hepatic steatosis of hepatic steatosis. Targeted intervention against ITGB3 to modulate CD36‐mediated lipid uptake may represent a novel therapeutic strategy for the treatment of MASH.

## Graphical abstract

In MASH, ITGB3 recruits LYN and drives its ubiquitin‐proteasomal degradation via phosphorylation. This relieves DHHC5 inhibition, enabling ITGB3/DHHC5/CD36 complex assembly to enhance CD36 palmitoylation and fatty acid uptake, thereby exacerbating disease. Targeting ITGB3 blocks this pathogenic axis and ameliorates MASH.

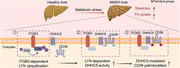

## 1 Introduction

Metabolic dysfunction‐associated steatotic liver disease (MASLD) has emerged as a major global health concern, affecting over 30% of the adult population and contributing to significant morbidity and mortality.^[^ ^1^ ^]^ Its progressive inflammatory subtype, metabolic dysfunction‐associated steatohepatitis (MASH), is pathologically defined by hepatocellular steatosis, ballooning degeneration, lobular inflammation, and varying degrees of fibrosis.^[^ ^2^ ^]^ Approximately 20–30% of MASLD patients develop MASH, a transition that substantially elevates the risk of cirrhosis, hepatocellular carcinoma (HCC), and liver failure, placing a heavy burden on healthcare systems worldwide.^[^ ^3^ ^]^ Despite this, pharmacologic options remain limited. The recent FDA approval of the thyroid hormone receptor‐β agonist Resmetirom (Rezdiffra) marks a critical milestone. However, in its pivotal trials, comprehensive histological responses—defined by the resolution of steatosis, the reduction of lobular inflammation, and crucially, a ≥1‐stage improvement in fibrosis without worsening of MASH—were achieved in only a subset (≈25‐30%) of treated patients.^[^ ^4^ ^]^ Complementing this, semaglutide, a GLP‐1 receptor agonist, has also shown efficacy in improving liver histology in MASLD, primarily through its systemic metabolic benefits.^[^ ^5^ ^]^ However, its relative lack of a direct anti‐fibrotic effect, as evidenced by not meeting the fibrosis endpoint in its phase 3 trial, highlights the distinct pathophysiological processes governing metabolic health versus fibrosis progression.^[^ ^5^ ^]^ This underscores the urgent need to further investigate the disease‐specific pathways causatively linked to MASH pathogenesis and identify potential therapeutic targets.

Accumulating evidence shows that components of extracellular matrix (ECM), in addition to their scaffolding function, contributes to liver pathology. Increased deposition of ECM and associated aberrant transmembrane signaling propagates liver fibrosis, carcinogenesis, and metabolic reprogramming.^[^ ^6^, ^7^, ^8^, ^9^, ^10^ ^]^ ECM remodeling modulates hepatocellular function via mechanotransduction and receptor‐mediated signaling, thereby influencing metabolic homeostasis.^[^ ^11^, ^12^, ^13^, ^14^ ^]^ Specific ECM components have been shown to alter lipid accumulation in hepatocytes, while matrix stiffness activates YAP‐dependent lipogenic transcriptional programs.^[^ ^14^ ^]^ These findings point to an underappreciated role of ECM components in regulating hepatic lipid metabolism in MASH pathogenesis.

Integrins, a family of heterodimeric transmembrane receptors, orchestrate bidirectional signaling between cells and the ECM.^[^ ^15^ ^]^ Among integrins, integrin β3 (ITGB3) has been extensively studied owing to its canonical roles in cellular processes including adhesion, endocytosis, survival signaling, angiogenesis and proliferation, with targeted therapeutics clinically applied to cardiovascular, neoplastic, and ophthalmic disorders.^[^ ^16^, ^17^, ^18^, ^19^ ^]^ Of note, we have recently shown a role for ITGB3 in temporal regulation of RhoA activation in renal epithelial cells.^[^ ^20^ ^]^ ITGB3 is established as a mediator of hepatic stellate cells (HSCs) activation and an imaging biomarker of fibrotic progression in the liver.^[^ ^21^, ^22^ ^]^ Although, ITGB3 has been shown to regulate endothelial cell injury and localized lipid accumulation in the vascular wall during atherogenesis by mediating cell adhesion and survival signaling pathways,^[^ ^23^ ^]^ the existing research remains confined to these classical mechanistic paradigms. Critically, whether ECM remodeling modulates hepatocellular lipid metabolism via ITGB3 and specifically through its non‐canonical membrane‐scaffolding function represents an unexplored frontier in MASH pathogenesis.

Here, we have identified ITGB3 as a key molecule that senses the ECM and is consistently upregulated in human liver tissue affected by MASH, as well as in multiple murine models of MASH. Our functional studies, involving both gain‐ and loss‐of‐function approaches, demonstrate that overexpressing ITGB3 specifically in hepatocytes worsens hepatic steatosis, inflammation, fibrosis, and systemic insulin resistance. Conversely, deleting ITGB3 specifically in hepatocytes confers protection in metabolic disease. Mechanistically, we have discovered that in response to metabolic stress, ITGB3 modulates the interaction between DHHC5 and CD36 which stabilizes DHHC5 activity, and facilitates the palmitoylation and plasma membrane localization of CD36, —thereby enhancing fatty acid (FA) uptake. ITGB3 also recruits LYN and promotes its proteasomal degradation via Y397 phosphorylation. The resultant loss of LYN relieves its inhibitory effect on DHHC5, which in turn enhances the functional output of the ITGB3/DHHC5/CD36 complex. Furthermore, both genetic silencing and pharmacological inhibition of ITGB3 using cyclic‐RGDfk significantly attenuate hepatic steatosis and fibrosis in MASH models. In summary, our findings define ITGB3 as a novel dual‐function effector that regulates hepatic lipid uptake and fibrosis in MASH under metabolic stress. Inhibition of ITGB3 could be a promising therapeutic target for MASH.

## 2 Results

### 2.1 ITGB3 Expression is Elevated in Human and Mouse MASH Livers

**Figure 1:**
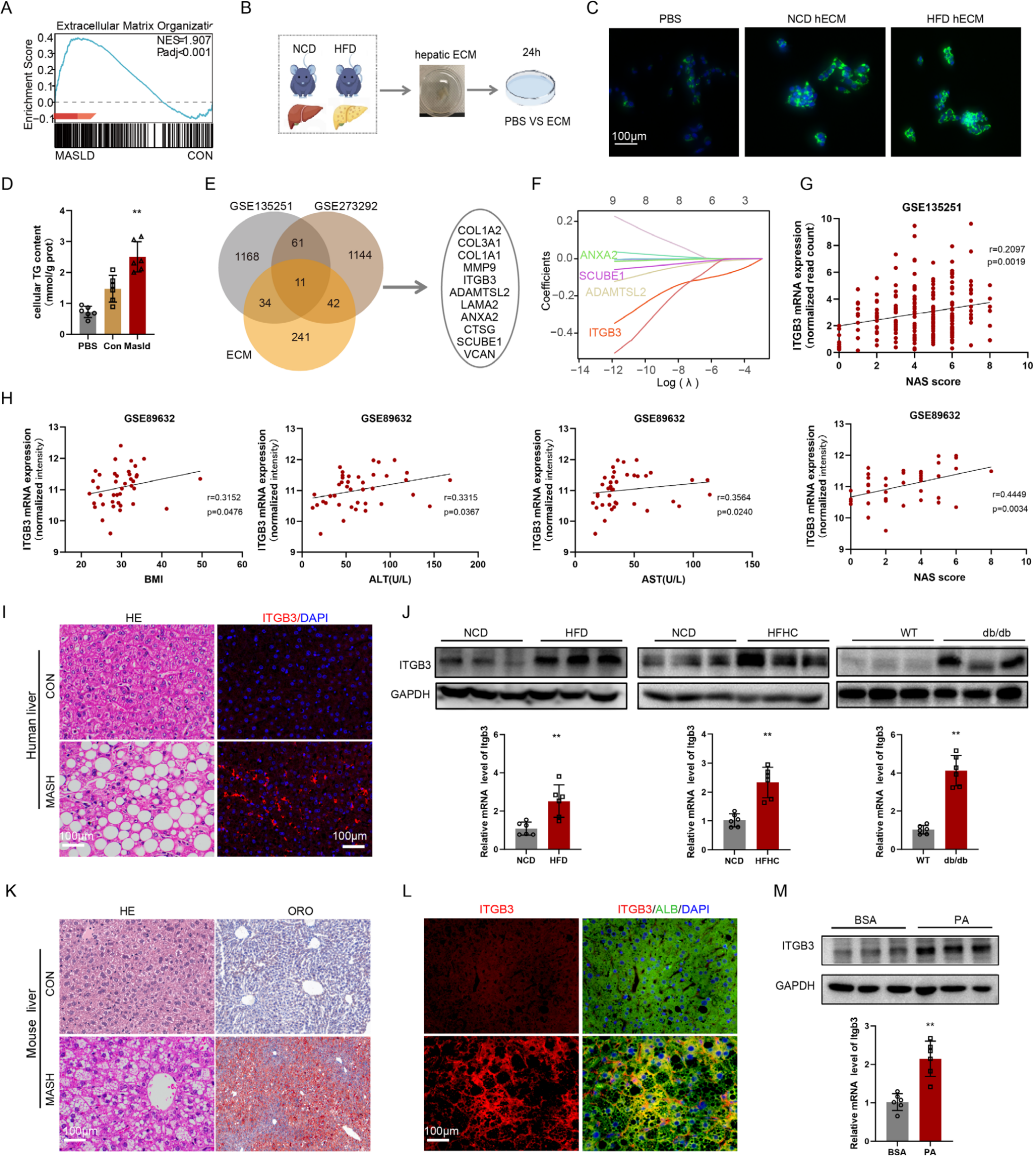
ITGB3 expression is upregulated in livers of Human and mouse MASH. A) Gene set enrichment analysis of GSE135251 demonstrated that upregulated genes in livers from the MASH group were enriched in ECM‐related pathways. B) Schematic diagram illustrating treatment of AML‐12 cells with hECM extracted from mice fed a normal chow diet (NCD) or high‐fat diet (HFD). C,D) Representative bodipy staining images (C) and intracellular triglyceride (TG) levels (D) in AML‐12 cells following exposure to NCD‐hECM or HFD‐hECM (n = 4). Scale bar, 100µm. E) Venn diagram showing altered ECM factors identified in RNA‐seq datasets (GSE135251 and GSE273292) from different fatty liver models. F) Results of LASSO regression analysis, showing that ITGB3 as a top predictor of MASH with variable coefficients across λ values. G,H) Correlation of ITGB3 expression with clinical indices in GEO datasets. I) Representative H&E staining and immunofluorescent staining for ITGB3 in liver tissues from healthy controls (HC, n = 10) and patients with metabolic dysfunction‐associated steatohepatitis (MASH, n = 18). Red fluorescence indicates ITGB3 protein. Scale bars, 100µm. J) Hepatic ITGB3 mRNA and protein levels in wild‐type mice fed an NCD or HFD for 16 weeks, a high‐fat/high‐cholesterol (HFHC) diet for 24 weeks, or in db/db^−^/^−^ mice and lean controls for 16 weeks (n = 6). K) Representative H&E and Oil Red O staining of liver sections from NCD and HFD mice (n = 6). Scale bar, 100µm. L) Immunofluorescent staining for albumin (ALB) and ITGB3 in livers from NCD and HFD mice, with DAPI counterstaining for nuclei (n = 6). Scale bar, 100µm. M) Following 12 h of starvation, HepG2 cells were treated with bovine serum albumin (BSA; 200µM)‐conjugated palmitic acid (PA; 300µM) for 24 or 48 h. Subsequently, the mRNA and protein expression levels of ITGB3 were quantified in these treated HepG2 cells (n = 6). The data are presented as the means ± SDs (**p <0.01,). Statistical analysis was performed with a two‐tailed Student's *t* test.

To investigate the contribution of the ECM to MASH pathogenesis, we first analyzed the publicly available transcriptomic dataset GSE135251. Gene set enrichment analysis (GSEA) revealed a significant upregulation of ECM‐associated gene signatures in liver tissues from MASH patients (**Figure** **1**A). To determine whether ECM remodeling contributes to hepatic lipid metabolism, hepatic ECM (hECM) was isolated from mice fed a HFD or normal control diet (NCD) for 16 weeks, and subsequently applied to AML‐12 hepatocytes in vitro (Figure 1B). The hECM derived from HFD‐fed mice markedly increased intracellular lipid accumulation (Figure 1C) and cellular triglyceride (TG) levels (Figure 1D) compared to hECM from NCD‐fed controls, indicating that HFD‐induced ECM remodeling promotes hepatic lipid deposition.

To identify ECM‐related genes mechanistically involved in hepatic steatosis, we performed integrative transcriptomic analyses across multiple human and murine MASH datasets (Figure 1E). Machine learning prioritization pinpointed ITGB3 as a core ECM‐linked gene most strongly associated with MASH progression (Figure 1F). In agreement with this, analysis of genome‐wide association study (GWAS) data from an open‐source GWAS database (*cvd.hugeamp.org*) revealed that ITGB3 exhibited the highest metabolic trait score among ECM‐related candidates (Table S3, Supporting Information). We further evaluated ITGB3 expression across multiple human MASH datasets, where ITGB3 mRNA levels positively correlated with both disease severity and obesity metrics (Figure 1G,H).

To further validate these findings, we performed immunofluorescence staining of liver biopsies from healthy individuals and patients with histologically confirmed MASH. These analyses demonstrated markedly elevated ITGB3 protein expression in MASH livers (Figure 1I). Congruently, in multiple mouse models of MASH including HFD‐fed, high‐fat high‐cholesterol (HFHC)‐fed, and leptin receptor deficient obese/diabetic db/db mice, both hepatic ITGB3 mRNA and protein levels were markedly upregulated (Figure 1J) and positively correlated with characteristic histopathological changes in liver (Figure 1K). Among the liver cell types, the heightened expression of ITGB3 was specifically co‐localized to hepatocytes as evidenced by co‐staining with the hepatocyte marker albumin (ALB) (Figure 1L). Additionally, in vitro treatment of HepG2 hepatocytes with palmitic acid (PA), a lipotoxic agent, significantly induced ITGB3 mRNA and protein expression (Figure 1M).

Collectively, these data from human tissues, multiple animal models, and in vitro systems robustly demonstrate that hepatic ITGB3 expression is consistently upregulated in MASH, and that its expression correlates with both lipid accumulation and disease severity, positioning ITGB3 as a candidate mediator in MASH pathogenesis.

### 2.2 ITGB3 Overexpression Exacerbates HFD–Induced Hepatic Steatosis

**Figure 2:**
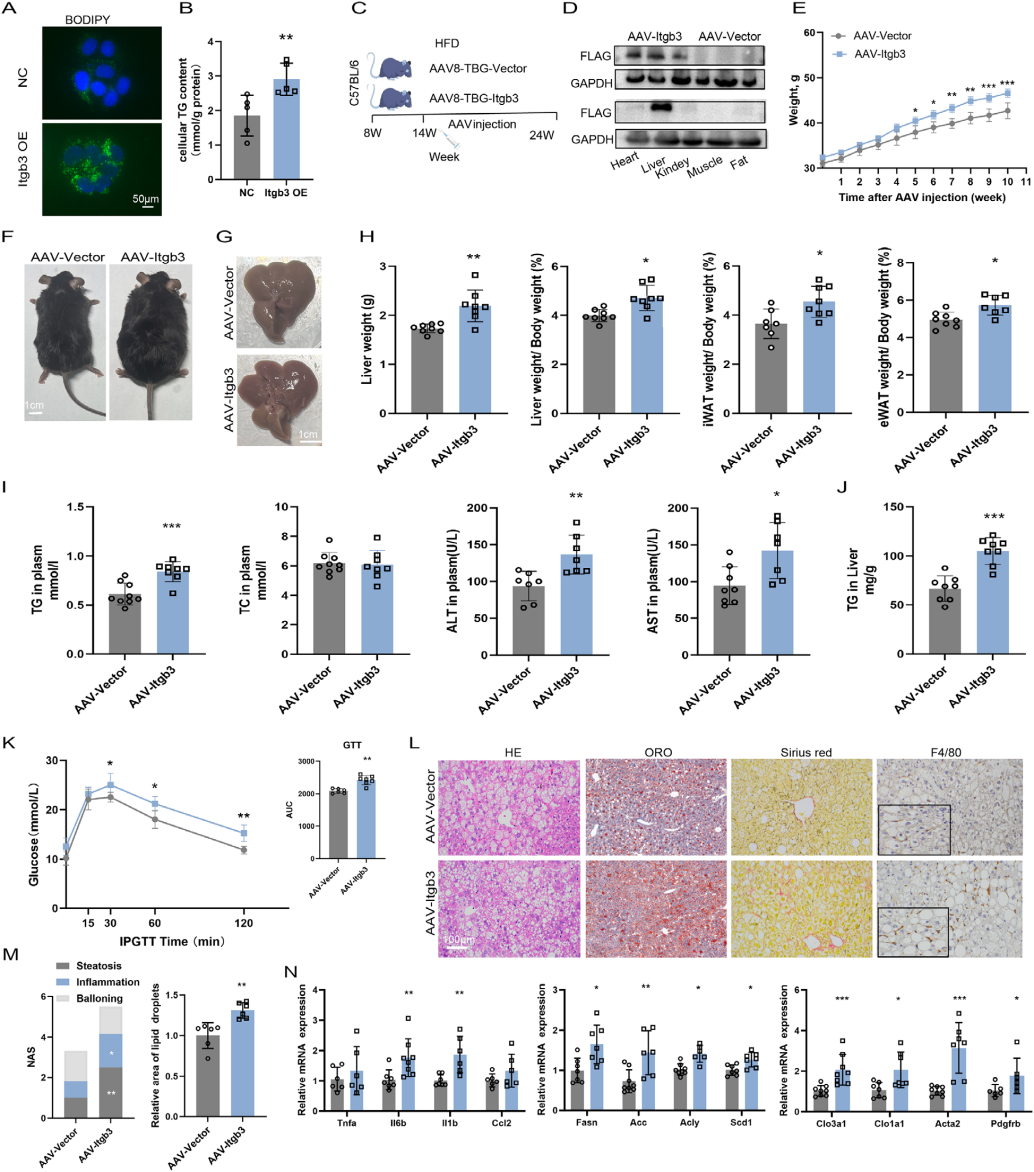
Hepatocellular ITGB3 overexpression promotes HFD‐induced hepatic steatosis. A,B) Representative bodipy staining images (A) and intracellular TG levels (B) in HepG2 cells transfected with empty vector (NC) or ITGB3‐overexpressing constructs (n = 5). Scale bar, 50µm. C) Schematic of the study design for adeno‐associated virus (AAV)‐mediated ITGB3 overexpression in C57BL/6J mice. The mice were fed an HFD for 6 weeks, then intravenously injected with AAV encoding ITGB3, and maintained on an HFD for an additional 10 weeks. D) Immunoblot analysis of hepatic ITGB3 protein levels, with GAPDH as a loading control (n = 3). E) Body weights of mice (n = 8). F,G) Representative photographs of mice (F) and livers (G) (n = 8). Scale bar, 1 cm. H) Liver weight, liver‐to‐body weight ratio, inguinal white adipose tissue (iWAT)‐to‐body weight ratio, and epididymal white adipose tissue (eWAT)‐to‐body weight ratio (n = 7–8). I) Serum levels of TG, total cholesterol (TC), alanine transaminase (ALT), and aspartate transaminase (AST) (n = 7‐8). J) Hepatic TG content (n = 7‐8). K) Glucose tolerance tests (GTTs) performed 8 weeks after AAV injection (n = 6). L) H&E, Oil Red O, Sirius Red, and F4/80 staining of liver sections (n = 6). Scale bar, 100µm. M) NAFLD Activity Score (NAS) and lipid accumulation area (n = 6). N) Relative mRNA levels of genes involved in lipid metabolism, inflammation, and fibrosis (n = 6–8). The data are presented as the means ± SDs (*p < 0.05, **p < 0.01, ***p < 0.001). Statistical analysis was performed with a two‐tailed Student's *t* test.

To elucidate the role of ITGB3 in hepatocellular lipid accumulation, we first conducted in vitro gain of function studies using HepG2 cells. Transfection with an ITGB3 overexpression plasmid led to a pronounced increase in intracellular lipid droplet accumulation, as visualized by BODIPY staining (**Figure** **2**A), alongside a significant elevation in intracellular TG content (Figure 2B). These findings suggest a direct role for ITGB3 in promoting hepatocyte lipid storage.

We next investigated the in vivo impact of hepatocyte‐specific ITGB3 overexpression using an adeno‐associated virus (AAV) vector system. Eight‐week‐old C57BL/6J mice were fed a HFD for 6 weeks, followed by tail vein injection of AAV8‐TBG‐ITGB3 or AAV8‐TBG‐Vector, and continued on HFD for an additional 10 weeks (Figure 2C,D). ITGB3‐overexpressing mice exhibited significantly greater body weight gain (Figure 2E,F), increased liver weight, and elevated liver‐to‐body weight ratio compared to control mice (Figure 2G,H). In parallel, expansion of both epididymal (eWAT) and inguinal (iWAT) white adipose tissue depots was observed (Figure 2H), indicative of systemic lipid dysregulation.

Biochemical analyses revealed that ITGB3 overexpression markedly increased serum TG, alanine aminotransferase (ALT), aspartate aminotransferase (AST), and hepatic TG levels (Figure 2I,J), highlighting both hepatocellular injury and lipid overload. Additionally, ITGB3‐overexpressing mice displayed impaired glucose tolerance relative to controls (Figure 2K), reflecting broader metabolic dysfunction.

ITGB3‐overexpressing mice displayed aggravated liver steatosis, evidenced by H&E and Oil Red O staining (Figure 2L,M). Mice with ITGB3‐overexpressing hepatocytes demonstrated elevated collagen deposition, as evidenced by Sirius red staining (Figure 2L). Furthermore, when compared to controls, ITGB3 overexpressing mice showed higher infiltration of proinflammatory myeloid cells in the liver analyzed by characteristic macrophage marker F40/80 (Figure 2L). These phenotypic changes were accompanied by transcriptional upregulation of genes involved in lipid metabolism (*Fasn, Acc, Acly, Scd1*) inflammatory signaling (*Tnfa, Il6b, Il1b, Ccl2*), and fibrogenesis (*Clo3a1, Clo1a1, Acta2, Pdgfrb*) (Figure 2N), further corroborating the pathological impact of ITGB3 activation in the liver.

These findings demonstrate that ITGB3 overexpression in hepatocytes not only potentiates hepatic steatosis and systemic metabolic dysregulation, but also amplifies hepatic inflammation and fibrotic remodeling, thereby expanding the known pathological repertoire of ITGB3 beyond its established role in fibrogenesis to include direct regulation of hepatic lipid metabolism.

### 2.3 Hepatocyte‐Specific Knockout of ITGB3 Ameliorates Hepatic Steatosis, Inflammation, and Fibrosis in HFD and HFHC Diet‐Induced Mouse Models

Given that ITGB3 overexpression exacerbated hepatic steatosis and fibrosis, we next investigated whether hepatocyte‐specific deletion of ITGB3 could mitigate these pathological features in MASH. To this end, we generated mice with hepatocyte‐specific deletion of ITGB3 (HKO) by crossing ITGB3^flox/flox^ mice with hepatocyte specific Albumin‐Cre mice and their corresponding control (FLOX) littermates (Figure S1A, Supporting Information) to evaluate the impact of ITGB3 deficiency on metabolic and pathological outcomes.

Under NCD conditions, no significant differences were observed between HKO and FLOX mice in terms of body weight, liver weight (Figure S1B, Supporting Information), serum TG, hepatic TG (Figure S1C, Supporting Information), ALT, and AST levels (Figure S1D, Supporting Information). These results suggest that ITGB3 deficiency does not alter basal metabolic phenotypes, indicating that ITGB3's role in lipid metabolism and inflammation is context‐dependent, particularly under pathological conditions.

**Figure 3:**
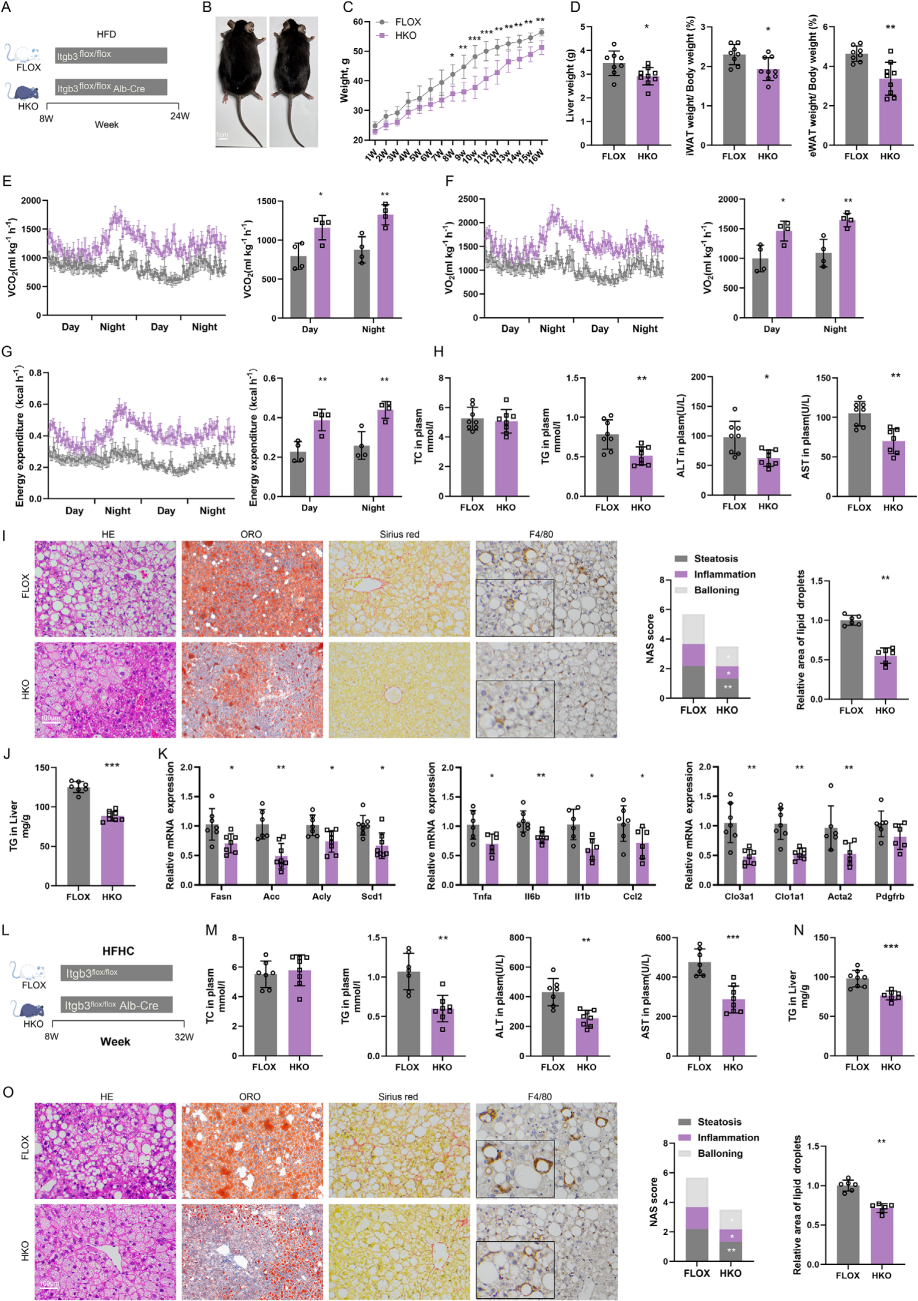
Hepatocyte‐specific ITGB3 knockdown ameliorates HFD and HFHC diet‐induced MASH. A) Schematic of the HFD‐induced model. B) Representative photographs of mice. Scale bar, 1 cm. C) Body weights (n = 7‐8). D) Liver weight, iWAT‐to‐body weight ratio, and eWAT‐to‐body weight ratio (n = 7‐8). E‐G) Metabolic parameters measured in metabolic cages during day and night cycles: arbon dioxide production (VCO_2_) (E), oxygen consumption (VO_2_) (F), and heat generation (G) (n = 4). H) Serum levels of TC, TG, ALT, and AST (n = 7–8). I) H&E, Oil Red O, Sirius Red, and F4/80 staining of liver sections (n = 6). Scale bar, 100µm. J) Hepatic TG content (n = 7‐8). K) Relative mRNA levels of genes involved in lipid metabolism, inflammation, and fibrosis (n = 6‐8). L) Schematic of the HFHC‐induced model. M) Serum levels of TC, TG, ALT, AST, and hepatic TG content (n = 7‐8). O) H&E, Oil Red O, Sirius Red, and F4/80 staining of liver sections (n = 6). Scale bar, 100µm. The data are presented as the means ± SDs (*p < 0.05, **p < 0.01, ***p < 0.001). Statistical analysis was performed with a two‐tailed Student's *t* test.

To examine the effect of ITGB3 deficiency in the context of MASH, we utilized two distinct mouse models. First, HKO and FLOX mice were fed a HFD for 16 weeks to induce hepatic steatosis and inflammation (**Figure** **3**A). After 8 weeks of HFD feeding, HKO mice exhibited significantly lower body weight compared to FLOX mice (Figure 3B,C). Additionally, HKO mice demonstrated reduced liver weight, and both eWAT and iWAT white adipose tissue ratios (Figure 3D; Figure S2A, Supporting Information). These mice also showed improved insulin sensitivity as evidenced by better glucose tolerance (Figure S2B, Supporting Information). Metabolic parameters including carbon dioxide production (Figure 3E), oxygen consumption (Figure 3F) and thermogenesis (Figure 3G) were higher in HKO mice, and no perceptible difference in terms of food and water intake was detected (Figure S2C, Supporting Information). Serum and liver biochemical analyses revealed a significant decrease in TG, ALT, and AST levels in HKO mice, indicating less hepatic lipid accumulation and reduced liver injury (Figure 3H–J). Histological examination further confirmed marked reductions in hepatic lipid accumulation, inflammatory cell infiltration, and fibrosis in HKO livers (Figure 3I). Furthermore, transcriptional analysis of liver tissue showed significant downregulation of genes involved in lipid metabolism, inflammation, and fibrosis (Figure 3K). Together, these results demonstrate that ITGB3 deficiency ameliorates HFD‐induced hepatic steatosis, fibrosis, inflammation, and metabolic deregulation.

To further elucidate the protective effects of ITGB3 deficiency on MASH, we employed the HFHC diet model (Figure 3L), which induces more pronounced liver injury and better mimics the histopathological and metabolic features of human MASH.^[^ ^24^ ^]^ HKO mice fed the HFHC diet for 24 weeks exhibited significantly attenuated body weight, liver weight, and fat mass compared to control FLOX mice (Figure S2D,E, Supporting Information). These mice also showed milder insulin resistance (Figure S2F, Supporting Information) and lower circulating lipid levels. Hepatic lipid accumulation was notably reduced, with significantly lower serum TG, hepatic TG levels, and serum ALT and AST (Figure 3M,N). Histological analysis revealed that ITGB3 deficiency effectively ameliorated HFHC diet‐induced hepatic steatosis, inflammation, and fibrosis (Figure 3O).

These findings provide robust in vivo evidence that hepatocyte‐specific ITGB3 deficiency improves the metabolic, inflammatory, and fibrotic features of MASH induced by both HFD and HFHC models. These results highlight the critical role of ITGB3 in regulating lipid metabolism and fibrosis in MASH pathogenesis.

### 2.4 ITGB3 Regulates Hepatic Lipid Uptake via DHHC5/CD36

**Figure 4:**
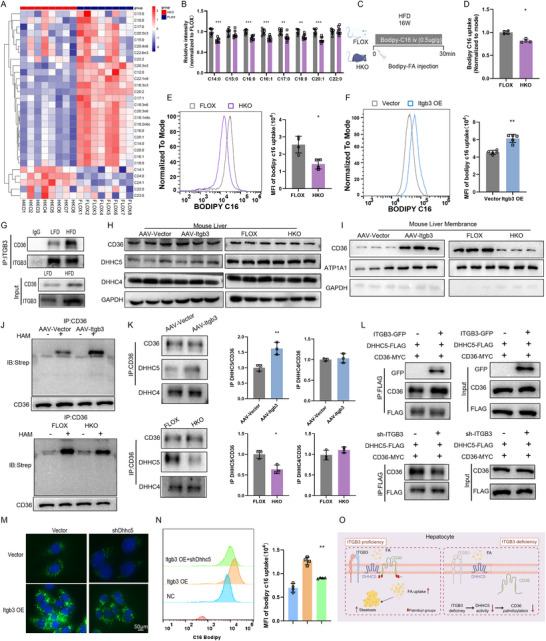
ITGB3 promotes long‐chain fatty acid uptake and CD36 palmitoylation via DHHC5. A,B) Heatmap (A) and quantitative analysis (B) of lipid‐targeted metabolomics in liver tissues from ITGB3‐floxed (FLOX) and hepatocyte‐specific ITGB3‐knockout (HKO) mice fed an HFD for 16 weeks (n = 8). C) Schematic illustrating the intraperitoneal injection of BODIPY‐labeled fatty acids to assess hepatic fatty acid uptake. D) Comparison of BODIPY‐C16 uptake in livers from FLOX and HKO mice (n = 4). E) Comparison of BODIPY‐C16 uptake in primary hepatocytes isolated from FLOX and HKO mice (n = 4). F) Comparison of BODIPY‐C16 uptake in HepG2 cells with ITGB3 overexpression (n = 4). G) Co‐immunoprecipitation (co‐IP) analysis of ITGB3‐interacting proteins in liver lysates. H) Representative immunoblots showing total hepatic CD36 protein levels (n = 6). I) Representative immunoblots showing CD36 protein levels in plasma membrane fractions isolated from liver tissues. J) Representative immunoblots showing CD36 palmitoylation in liver tissues (n = 4). K) Co‐IP analysis of interactions between CD36 and DHHC4 or DHHC5 in liver lysates (n = 4). L) Co‐IP assay in HepG2 cells cotransfected with FLAG‐DHHC5 and MYC‐CD36 to evaluate the DHHC5‐CD36 interaction with or without ITGB3 overexpression. M) Comparison of BODIPY‐C16 uptake in HepG2 cells treated with FLAG‐ITGB3 or shDHHC5 (n = 6). Scale bar, 50µm. N) Comparison of BODIPY‐C16 uptake in HepG2 cells treated with FLAG‐ITGB3 or shDHHC5 (n = 4). O) Schematic summary of the proposed mechanism. The data are presented as the means ± SDs (*p < 0.05, **p < 0.01, ***p < 0.001). The data in N were statistically analysed with one‐way ANOVA, and the data in the other panels were analysed with a two‐tailed Student's *t* test.

We next focused on elucidating the underlying mechanisms by which ITGB3 modulates lipid metabolism. Metabolomic analysis of liver tissues from HFD‐fed HKO and FLOX mice revealed significantly lower levels of long‐chain fatty acids (LCFAs) in HKO livers compared to FLOX controls (**Figure** **4**A,B). Given that hepatic FA accumulation is largely driven by FA uptake rather than *de novo* lipogenesis (DNL) in MASH, we used BODIPY C16, a fluorescent FA tracer, to assess hepatic FA uptake in vivo (Figure 4C). HKO mice exhibited reduced hepatic long‐chain FA uptake compared to FLOX mice (Figure 4D,E), confirming the importance of ITGB3 in hepatic lipid uptake.

To complement these in vivo observations, in vitro studies were performed in hepatocytes. ITGB3 overexpression in HepG2 cells led to a significant increase in long‐chain FA uptake (Figure 4F), supporting the hypothesis that ITGB3 directly modulates hepatic lipid metabolism.

CD36 is a well‐established mediator of long‐chain FA uptake in the liver, and its function is highly dependent on both its expression and localization to the plasma membrane.^[^ ^25^, ^26^ ^]^ Co‐immunoprecipitation (Co‐IP) assays revealed a direct interaction between ITGB3 and CD36, which was notably enhanced in response to HFD feeding (Figure 4G). These findings were corroborated by in vitro experiments in HepG2 cells (Figure S3A, Supporting Information). Interestingly, although ITGB3 overexpression did not alter the total levels of CD36 protein, it increased CD36 membrane localization (Figure S3B,C, Supporting Information). This observation was confirmed in livers of both ITGB3‐overexpressing and HKO mice (Figure 4H,I), suggesting that ITGB3 plays a key role in regulating the membrane trafficking of CD36.

Given the critical role of palmitoylation in the membrane localization of CD36, we assessed CD36 palmitoylation levels using the acyl‐biotin exchange (ABE) assay on liver proteins.^[^ ^27^ ^]^ ITGB3 overexpression resulted in increased CD36 palmitoylation, whereas ITGB3 deficiency significantly reduced hepatic CD36 palmitoylation (Figure 4J; Figure S3D, Supporting Information), reinforcing the hypothesis that ITGB3 regulates CD36 membrane trafficking through palmitoylation.

To investigate the specific palmitoyltransferase involved in this process, we performed Co‐IP assays to examine interactions between CD36 and palmitoyltransferases DHHC4 and DHHC5.^[^ ^26^ ^]^ Our results demonstrated that ITGB3 overexpression enhanced the interaction between CD36 and DHHC5, but did not affect CD36‐DHHC4 binding. In contrast, ITGB3 deficiency weakened the CD36‐DHHC5 interaction without altering the CD36‐DHHC4 binding (Figure 4K). Concordant results were obtained in HepG2 cells co‐overexpressing DHHC5 and CD36, which confirmed that ITGB3‐mediated regulation of CD36 membrane localization involves DHHC5 (Figure 4L).

To further evaluate the functional impact of DHHC5 in ITGB3‐mediated lipid metabolism, we silenced DHHC5 in HepG2 cells overexpressing ITGB3. Silencing DHHC5 (siDHHC5) significantly reduced CD36 membrane localization and largely reversed the increase in CD36 membrane localization induced by ITGB3 overexpression (Figure S3E, Supporting Information). Lipid droplet staining and flow cytometry analysis further showed that DHHC5 silencing attenuated lipid accumulation and lipid uptake caused by ITGB3 overexpression (Figure 4M,N).

Together, these findings indicate that ITGB3 regulates hepatic lipid uptake through a DHHC5‐mediated mechanism, where DHHC5 facilitates CD36 palmitoylation and its subsequent membrane localization (Figure 4O), underscoring the role of ITGB3 as a key modulator of lipid metabolism in hepatocytes.

### 2.5 Restoring DHHC5 Expression in HKO Mice is Sufficient to Promote Hepatic Steatosis

**Figure 5:**
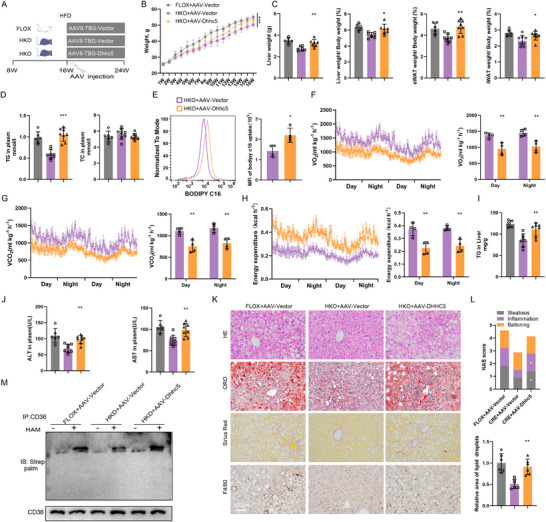
DHHC5 replenishment restores HFD‐induced hepatic steatosis in HKO Mice. A) Schematic of the experimental design. B) Body weights (n = 7‐8). C) Liver weight, liver‐to‐body weight ratio, iWAT‐to‐body weight ratio, and eWAT‐to‐body weight ratio (n = 7‐8). D) Serum levels of TG and TC. E) Comparison of BODIPY‐C16 uptake in primary hepatocytes from HKO mice injected with AAV‐DHHC5 or AAV‐vector. F‐H) Metabolic parameters measured in metabolic cages during day and night cycles: VO_2_ (F), VCO_2_ (G), and heat generation (H) (n = 4). I) Hepatic TG content (n = 7‐8). J) Serum ALT and AST levels (n = 7‐8). K) H&E, Oil Red O, Sirius Red, and F4/80 staining of liver sections (n = 6). Scale bar, 100µm. L) NAS and lipid accumulation area (n = 6). M) Representative immunoblots showing CD36 palmitoylation in liver tissues (n = 4). The data are presented as the means ± SDs (*p < 0.05, **p < 0.01, ***p < 0.001). The data in (E, F, G and H) were statistically analysed with a twotailed Student's *t* test, and the data in the other panels were analysed with one‐way ANOVA.

To further investigate the role of DHHC5 in hepatic lipid metabolism, we generated hepatocyte‐specific DHHC5‐overexpressing mice by administering AAV8‐TBG‐DHHC5 via tail vein injection to HKO and FLOX control mice (**Figure** **5**A). DHHC5 overexpression resulted in significantly increased body weight compared to HKO mice (Figure 5B), and was also associated with increased liver weight, liver‐to‐body weight ratio, and expansion of both eWAT and iWAT depots (Figure 5C).

Biochemical analysis revealed that DHHC5 overexpression led to a significant increase in serum TG levels, although TC levels remained unaffected (Figure 5D). Flow cytometry analysis demonstrated that DHHC5 overexpression enhanced lipid uptake in hepatocytes (Figure 5E), further supporting the role of DHHC5 in facilitating hepatic lipid accumulation. Additionally, DHHC5‐overexpressing mice exhibited lower VO2(Figure 5F), VCO2(Figure 5G), and overall energy expenditure (Figure 5H) compared to controls, indicating altered metabolic activity.

Histological and biochemical analyses revealed that DHHC5 overexpression significantly increased serum ALT and AST levels, as well as hepatic TG levels, indicative of hepatocellular injury and lipid overload (Figure 5I,J). Histological examination showed that the protective effects of ITGB3 deficiency against HFD‐induced hepatic steatosis, inflammation, and fibrosis were significantly reversed by restoring DHHC5 expression in HKO mice (Figure 5K,L), suggesting that DHHC5 upregulation abrogates the protective effects of ITGB3 deficiency on liver pathology.

At the molecular level, DHHC5 overexpression counteracted the reduction in CD36 palmitoylation caused by ITGB3 deficiency, as evidenced by increased CD36 palmitoylation in the livers of DHHC5‐overexpressing mice (Figure 5M). This finding further supports the hypothesis that ITGB3 facilitates lipid uptake through DHHC5‐mediated CD36 palmitoylation, highlighting the importance of DHHC5 in regulating lipid metabolism in hepatocytes. In summary, these findings demonstrate that ITGB3 regulation of lipid uptake is mediated through the DHHC5‐CD36 interactions.

### 2.6 ITGB3 Regulates the Activity of DHHC5 via LYN

**Figure 6:**
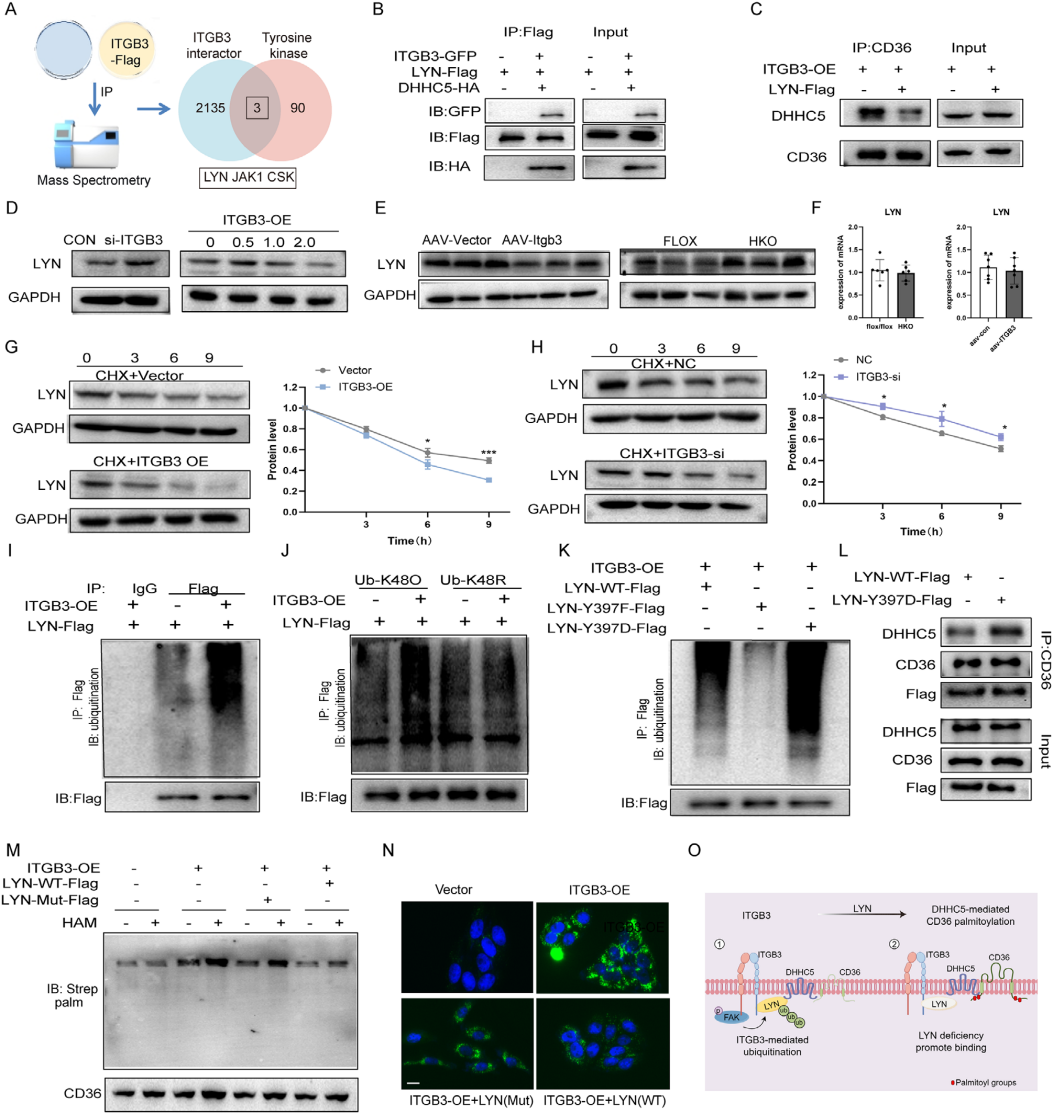
ITGB3‐mediated stabilization of LYN regulates CD36 palmitoylation and subsequent lipid uptake. A) Schematic of ITGB3‐binding protein identification. Venn diagram from LC‐MS/MS analysis showing that LYN is a common interacting partner of ITGB3 and DHHC5. B) Co‐IP assay in HepG2 cells confirming interactions of LYN with both ITGB3 and DHHC5. C) Co‐IP assay evaluating the CD36‐DHHC5 interaction with or without LYN overexpression. D) Representative immunoblots showing LYN protein levels in HepG2 cells transfected with NC, si‐ITGB3 (100 µM), empty vector, or ITGB3 (0.5, 1.0, or 2.0 ng). E) Representative immunoblots showing total hepatic LYN protein levels (n = 6). F) Relative hepatic LYN mRNA levels (n = 5). G,H) Representative immunoblots and quantification of LYN protein stability in HepG2 cells transfected with NC, si‐ITGB3 (100 µM), empty vector, or ITGB3, followed by cycloheximide (CHX 50 µg/mL) treatment for indicated time points. I) Representative immunoblots showing LYN ubiquitination with or without ITGB3 overexpression. J) Representative immunoblots showing LYN ubiquitination in cells transfected with HA‐ubiquitin (K48R) or HA‐ubiquitin (K48O). K) Representative immunoblots showing LYN ubiquitination in cells transfected with LYN‐WT, LYN‐Y397F, or LYN‐Y397D. L) Co‐IP assay assessing the CD36‐DHHC5 interaction in cells expressing LYN‐WT or LYN‐Y397D. M) Representative immunoblots showing CD36 palmitoylation in HepG2 cells transfected with ITGB3‐overexpression, LYN‐WT, or LYN‐Mut (Y397D) (n = 3). N) Representative bodipy staining images of HepG2 cells cotransfected with ITGB3‐overexpression, LYN‐WT, or LYN‐Mut (Y397D) (n = 3). Scale bar, 50 µm. O) Schematic summary of the proposed mechanism. The data are presented as the means ± SDs (*p < 0.05, ***p <0.001). Statistical analysis was performed with a two‐tailed Student's *t* test.

To elucidate the molecular mechanism by which ITGB3 regulates DHHC5 activity, we performed immunoprecipitation‐coupled mass spectrometry (IP‐MS) to identify proteins interacting with either ITGB3 or DHHC5. Among candidate interactors, LYN kinase—a member of the SRC family and a known regulator of palmitoyltransferase function—was identified as a shared binding partner (**Figure** **6**A).^[^ ^25^ ^]^ Subsequent in vitro validation confirmed that LYN physically associates with both ITGB3 and DHHC5, forming a ternary complex (Figure 6B). Importantly, LYN overexpression reduced DHHC5–CD36 binding (Figure 6C), suggesting that LYN acts as a negative regulator of this interaction.

To determine whether ITGB3 modulates LYN levels, we manipulated ITGB3 expression in HepG2 cells. ITGB3 knockdown increased LYN protein expression, whereas ITGB3 overexpression decreased it (Figure 6D). Interestingly, this regulation occurred post‐transcriptionally, as LYN mRNA levels remained unchanged (Figure 6E,F). Treatment of cells with cycloheximide (CHX) revealed that ITGB3 overexpression significantly reduced the half‐life of LYN, whereas ITGB3 silencing extended it (Figure 6G,H), indicating that ITGB3 controls LYN protein stability. To identify the degradation mechanism, cells were treated with the proteasome inhibitor MG‐132 or the lysosome inhibitor chloroquine (CQ). Only MG‐132 treatment rescued LYN degradation, suggesting that ITGB3 promotes proteasome‐dependent LYN degradation (Figure S5A, Supporting Information). Furthermore, ITGB3 enhances K48‐linked ubiquitination of LYN, a canonical signal for proteasomal degradation (Figure 6I,J).

Given that integrins can activate phosphorylation cascades, we investigated whether ITGB3‐mediated phosphorylation influenced LYN stability. We found that ITGB3 promotes the tyrosine phosphorylation of LYN via FAK signaling (Figure S5B, Supporting Information). Crucially, inhibition of LYN phosphorylation markedly increased its ubiquitination (Figure S5C, Supporting Information). Together, these results suggest that ITGB3‐mediated phosphorylation of LYN precedes and prevents its ubiquitination, thereby acting as a stabilizing modification.

To identify specific phosphorylation sites, we performed in silico analysis and identified Y397 and Y508 as putative regulatory residues (Figure S5D, Supporting Information). Mutation of Y397 to phenylalanine (Y397F) suppressed LYN ubiquitination, whereas Y508 had no effect (Figure S5E, Supporting Information), indicating that phosphorylation at Y397 is essential for LYN stability.

To functionally validate the role of LYN in DHHC5‐mediated lipid metabolism, we overexpressed either wild‐type (WT) LYN or the Y397D phospho‐mimetic mutant in hepatocytes. The Y397 mutant exhibited greater ubiquitination resistance and enhanced protein stability (Figure 6K; Figure S5F, Supporting Information). Co‐immunoprecipitation assays showed that Y397D‐LYN promoted DHHC5–CD36 binding (Figure 6L). Moreover, reintroduction of WT‐LYN in ITGB3‐overexpressing cells reduced CD36 palmitoylation and lipid accumulation, while Y397D mutant had no such effect (Figure 6M,N). These results indicate that LYN destabilization by ITGB3 is required for DHHC5–CD36 complex formation and downstream lipid uptake.

Taken together, our above data reveal that ITGB3 promotes LYN degradation via K48‐linked ubiquitination, thereby relieving LYN‐mediated repression of DHHC5. This post‐translational modulation of LYN by ITGB3 facilitates CD36 palmitoylation, enhances FA uptake, and supports the formation of the DHHC5–CD36 complex (Figure 6O), establishing LYN as a central regulatory node in the ITGB3/DHHC5/CD36 complex.

### 2.7 Therapeutic Approaches Targeting ITGB3 Improve MASH

Given the mechanistic and pathological evidence implicating ITGB3 as a central driver of MASH, we next evaluated its potential as a therapeutic target using both genetic and pharmacologic strategies.

**Figure 7:**
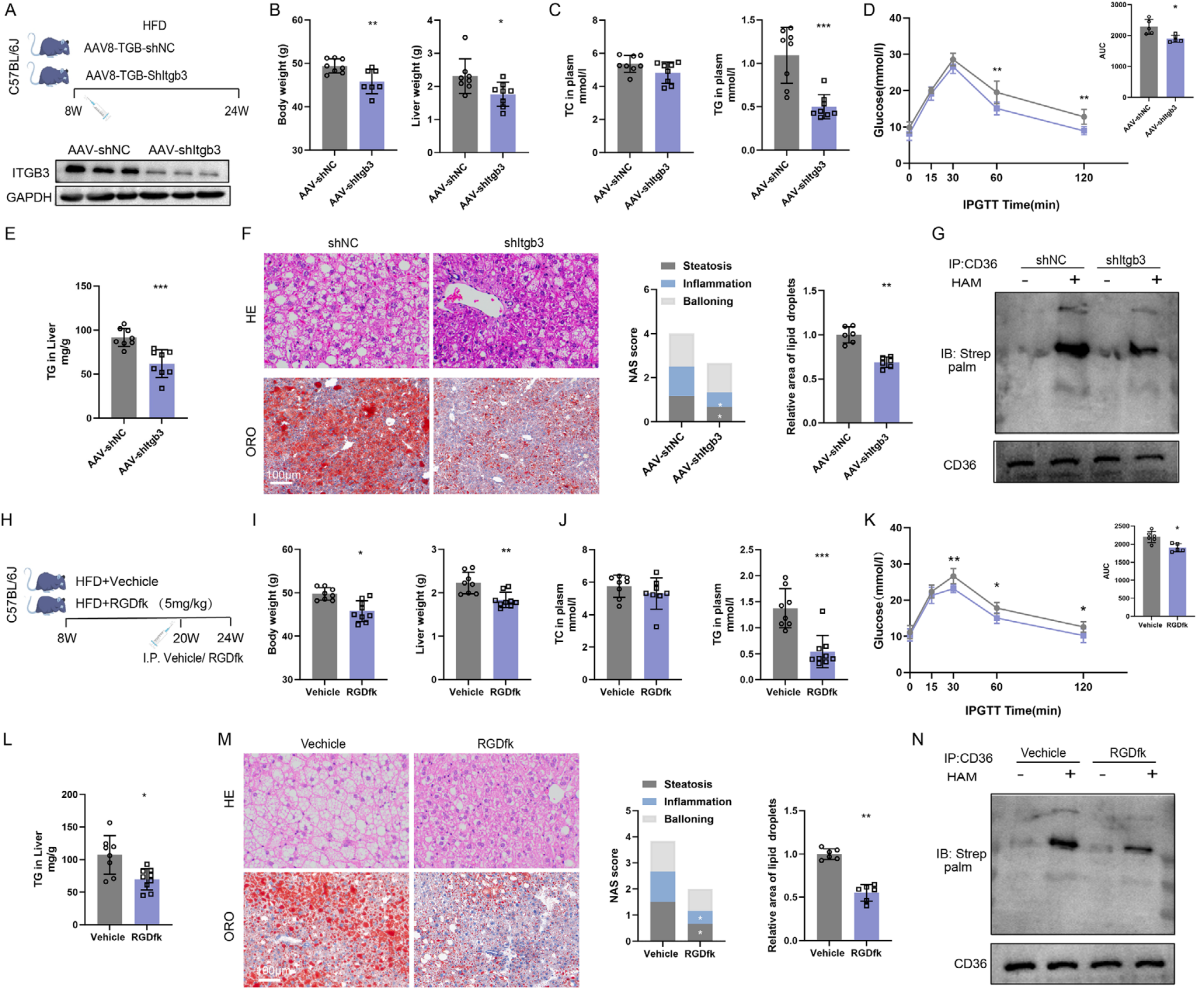
Therapeutic Targeting of ITGB3 Attenuates MASH. A) Schematic of the AAV‐shItgb3‐mediated knockdown model. B) Body weights and liver weights (n = 7‐8). C) Serum levels of TC and TG (n = 7‐8). D) GTT results (n = 5). E) Hepatic TG content (n = 7‐8). F) H&E and Oil Red O staining, NAS, and lipid accumulation area (n = 6). Scale bar, 100 µm. G) Representative immunoblots of CD36 palmitoylation in AAV‐shItgb3‐treated mouse liver tissues. (n = 4). H) Schematic of the cyclic‐RGDfk treatment model. I) Body weights and liver weights (n = 7‐8). J) Serum levels of TC and TG (n = 7‐8). K) GTT results (n = 5). L) Hepatic TG content (n = 7‐8). M) H&E and Oil Red O staining, NAS, and lipid accumulation area (n = 6). Scale bar, 100 µm. The data are presented as the means ± SDs (*p < 0.05, **p < 0.01, ***p < 0.001). Statistical analysis was performed with a two‐tailed Student's *t* test.

In the first approach, we employed AAV8‐mediated hepatocyte‐specific knockdown of ITGB3 (AAV8‐TBG‐shITGB3). C57BL/6J mice were injected via tail vein with AAV8‐shITGB3 and then maintained on a HFD (**Figure** **7**A). Compared to control mice, ITGB3 knockdown significantly improved body weight and liver weight, and also led to reductions in serum TG levels, improved glucose tolerance, and enhanced overall metabolic profiles (Figure 7B–E). Histological evaluation of liver sections revealed a marked reduction in hepatic steatosis in AAV8‐shITGB3‐treated mice (Figure 7F). This improvement was consistent with a significant reduction in CD36 palmitoylation observed at the molecular level (Figure 7G), thus demonstrating the mechanistic relevance of ITGB3 inhibition in preventing MASH onset and progression.

In the second approach, we utilized cyclic‐RGDfk, a synthetic peptide inhibitor that specifically blocks ITGB3 activity. C57BL/6J mice were administered oral cyclic‐RGDfk (1 or 5 mg/kg/day, dissolved in PBS) while being fed an HFD for 4 weeks. Treatment with cyclic‐RGDfk led to improvements in body weight, serum TG levels, and glucose tolerance (Figure S5A–D, Supporting Information), accompanied by histological improvement in hepatic steatosis (Figure S5I, Supporting Information). Furthermore, direct assessments of liver and kidney function showed no significant abnormalities at the dosage used, supporting its systemic safety (Figure S5E‐H, Supporting Information). To examine the long‐term effects of pharmacological ITGB3 inhibition, a second cohort of mice was fed an HFD for 16 weeks, with daily cyclic‐RGDfk treatment initiated during the final 4 weeks (Figure 7H). cyclic‐RGDfk administration during late‐stage after disease‐onset significantly attenuated HFD‐induced obesity, glucose intolerance, and dyslipidemia (Figure 7I–L). These changes were associated with a marked reduction in hepatic steatosis and inflammation, along with a parallel decrease in CD36 palmitoylation (Figure 7M,N).

These findings establish that both genetic silencing and pharmacological inhibition of ITGB3 ameliorates MASH and identify ITGB3 as disease‐specific target for the treatment of MASH.

## 3 Discussion

This study identifies ITGB3 as a key regulator of hepatic lipid metabolism and fibrosis in MASH through a novel ITGB3/DHHC5/CD36 complex. We demonstrate that ITGB3 assembles this complex and promotes LYN kinase destabilization, thereby enhancing DHHC5‐mediated CD36 palmitoylation and subsequent CD36‐dependent FA uptake. Hepatocyte‐specific ITGB3 overexpression exacerbated steatosis, inflammation, and fibrosis, whereas ITGB3 deletion or inhibition—via AAV‐shRNA or the cyclic‐RGDfk peptide—ameliorated these features across multiple murine MASH models. These findings position ITGB3 as a dual‐function effector, linking ECM signaling with lipid dysregulation, and highlight it as a promising therapeutic target for MASH.

Building on this concept of ECM‐driven pathogenesis, we note that dynamic alterations in ECM composition, stiffness, and signaling are critical determinants of hepatic cell fate.^[^ ^28^ ^]^ Recent studies have revealed that pathological ECM remodeling can activate intracellular metabolic sensors, such as the AMP‐activated protein kinase (AMPK) pathway, thereby influencing hepatocellular energy metabolism.^[^ ^29^, ^30^, ^31^ ^]^ Importantly, even in the early stages of MASLD, significant alterations in ECM structure and composition are already apparent. Remarkably, after therapeutic reversal of steatosis, residual ECM abnormalities persist, leading to continued hepatocyte injury, despite normalization of lipid content.^[^ ^13^ ^]^ This persistent remodeling of the ECM not only maintains hepatic inflammation and lipotoxic stress but also drives progression to metabolic HCC in advanced disease stages.^[^ ^31^ ^]^ Prior research has implicated that YAP‐driven transcriptional activation of lipogenic enzymes as a key mediator of ECM‐induced metabolic shifts.^[^ ^14^ ^]^ Building on these findings, we applied multi‐omics integration of transcriptomics, GWAS, and machine learning techniques to identify ITGB3 as the key ECM and lipids dual‐responsive effector in hepatocytes. This discovery addresses a significant gap in our understanding of how the interaction between ECM and lipid metabolism contributes to the progression of MASH.

ITGB3, an important member of the integrin family, has been implicated in fibrogenesis across multiple organs, including the liver.^[^ ^18^, ^20^, ^21^ ^]^ Our findings establish a critical role for hepatocyte‐autonomous ITGB3 signaling in driving MASH progression through a mechanism distinct from its well‐characterized function in hepatic stellate cells (HSCs). While previous studies have primarily focused on ITGB3 as a key mediator of HSC activation and extracellular matrix (ECM) remodeling—where it promotes fibrogenesis through SMAD2/3 activation and mechanical signaling—our work reveals a previously unappreciated, parenchymal mechanism.^[^ ^21^ ^]^ Specifically, we demonstrate that hepatocyte‐specific ITGB3 disrupts lipid uptake and insulin sensitivity, creating a pro‐inflammatory and pro‐fibrotic microenvironment that secondarily amplifies HSC activation. This hepatocyte‐driven pathway operates upstream of and in parallel to the established stromal functions of ITGB3. Whereas in HSCs, integrin signaling directly regulates collagen production and contraction, in hepatocytes it primarily governs metabolic homeostasis and cell survival. This cell‐type‐specific duality underscores the pleiotropic nature of integrin signaling in liver pathology and positions hepatocyte ITGB3 as a novel metabolic checkpoint.

Therapeutically, this distinction is crucial. Targeting hepatocyte ITGB3 could ameliorate steatosis, inflammation, and insulin resistance—core metabolic defects of MASH—while potentially avoiding the broad inhibition of stromal ITGB3 that might impair physiological ECM maintenance. While cyclic‐RGDfk is a well‐characterized ITGB3 antagonist with high affinity and specificity for its target, extensive in vivo studies and human data support its translational potential by showing no significant thrombocytopenia, bleeding risks, or impaired platelet function associated with its use or ITGB3 therapeutic inhibition.^[^ ^19^, ^32^, ^33^, ^34^ ^]^ We acknowledge that systemic administration of cyclic‐RGDfk may have extra‐hepatic effects beyond platelets, which were not comprehensively evaluated in the current study and warrant further investigation in future work. Future studies should further delineate the downstream effectors of hepatocyte versus stellate cell ITGB3 signaling to enable even more precise therapeutic targeting.

Our study elucidates a critical functional link among the four lipid metabolism‐related molecules—ITGB3, LYN, DHHC5, and CD36—that governs hepatic lipid homeostasis. CD36 plays a critical role in disrupting hepatic lipid homeostasis in MASH, with its palmitoylation and membrane localization directly governing the uptake of circulating fatty acids—the source of roughly 60% of hepatic triglycerides.^[^ ^35^, ^36^, ^37^, ^38^ ^]^ Recent studies have demonstrated that ITGB3 regulates smooth muscle cell cholesterol metabolism via the TLR4/CD36 signaling axis.^[^ ^39^ ^]^ Given this established role of ITGB3 in CD36‐associated metabolic regulation, whether ITGB3 further modulates hepatic FA uptake through CD36 stabilization warrants rigorous investigation. Our results show that ITGB3 assembles the LYN/DHHC5/CD36 complex, subsequently enhances CD36‐mediated FA uptake. Co‐immunoprecipitation (Co‐IP) assays performed in both in vitro and in vivo settings demonstrated that ITGB3 modulates the interaction between DHHC5 and CD36. Results from in vivo rescue experiments indicate that DHHC5, a key downstream effector of ITGB3, plays a critical role in hepatic lipid metabolism. LYN, a member of the SRC family, has been shown to regulate CD36 palmitoylation and the subsequent uptake of FA.^[^ ^35^, ^40^ ^]^ Similar to our hepatocyte‐specific ITGB3‐overexpressing mice, adipocyte‐specific LYN knockout cells exhibit increased DHHC5 activity and a subsequent elevation in CD36 palmitoylation.^[^ ^40^ ^]^ Although prior studies have established LYN's role in hepatic lipid metabolism, the upstream regulators influencing its activity under metabolic stress remained poorly understood. In this study, we establish ITGB3 as a novel intracellular regulator of LYN by demonstrating its direct binding and control over LYN stability, which subsequently modulates DHHC5 activity, thereby expanding the mechanistic framework of lipid regulatory pathways.

Interestingly, our findings reveal a non‐canonical, phosphorylation‐dependent degradation pathway. ITGB3 enhances the tyrosine phosphorylation of LYN at Y397, priming it for proteasomal degradation. Mutation of Y397, but not Y508, stabilized LYN and preserved its inhibitory effect on DHHC5/CD36 complex. This resolves existing conflicting reports; while LYN inhibitors have been shown to improve liver fibrosis and lipid metabolism,^[^ ^41^, ^42^ ^]^ other studies indicate LYN deficiency enhances CD36 palmitoylation in adipocytes.^[^ ^40^ ^]^ Our data clarify that LYN functions as a context‐dependent brake on hepatic lipid uptake, and that ITGB3–mediated LYN destabilization is a critical driver of steatogenic signaling in MASH.

Despite the strengths of this study, several limitations warrant consideration. First, while transcriptomic and machine learning analyses identified hepatocyte‐enriched ITGB3 as a key MASH effector, the roles of other ECM components and integrin subunits remain insufficiently explored. Broader dissection of the ECM–integrin–lipid signaling landscape is essential for a more integrated mechanistic framework. Second, while our data delineate a functional complex, further in vivo validation using conditional, inducible models is needed to confirm pathway causality. Finally, although both AAV‐mediated knockdown and cyclic‐RGDfk‐based inhibition showed therapeutic efficacy, future studies should evaluate the durability, safety, and off‐target effects of ITGB3‐targeted therapies across diverse MASH models and translational platforms. Addressing these gaps will be pivotal for clinical translation.

In conclusion, our study identifies a previously unrecognized ITGB3/DHHC5/CD36 complex that acts as a central mechanism governing hepatic lipid homeostasis in the context of MASH. This complex exerts its regulatory effects by promoting the palmitoylation and cell membrane localization of CD36 through LYN degradation and DHHC5 activity. Importantly, ITGB3 serves a dual function—integrating metabolic activation with fibrogenic signaling—making it a key regulatory node in the development of MASH. Our findings not only highlight a novel function of ITGB3 that is independent of its classic role as an ECM receptor in fibrosis process but also introduce an extracellular matrix‐driven mechanism of lipid metabolic reprogramming. This provides valuable insights into the underlying mechanisms and establishes a basis for targeted therapeutic approaches against MASH.

## 4 Experimental Section

### Human Liver Samples

Human liver specimens were obtained from individuals undergoing partial hepatectomy or liver transplantation. Each sample was independently assessed using the NAS Score, which evaluates MASLD activity by combining scores for hepatic steatosis, ballooning degeneration, and lobular inflammation. Patients with NAS >4 were diagnosed with MASH. Exclusion criteria comprised alcoholic liver disease and viral hepatitis. A total of 10 normal liver samples and 18 MASH liver samples were included for subsequent experiments. Clinical characteristics such as sex, height, age and BMI of each subject are summarized in Table S1 (Supporting Information). The collection of all human samples was approved by the Ethics Committee of Tongji Hospital, Tongji Medical College, Huazhong University of Science and Technology (Approval No: TJ‐IRB20210760).

### Animal Studies

All animal studies followed approved protocols by the Institutional Animal Care and Use Committee of Huazhong University of Science and Technology ([2024] IACUC: 4866). All data were from male mice. C57BL/6J wild‐type male mice were obtained from Cyagen Co. Ltd., Wuhan, China. ITGB3flox/flox and Albumin‐Cre (Alb‐Cre) were obtained from Cyagen Co. Ltd., Wuhan, China. All mice were housed in a pathogen‐free environment (22‐26 °C, 12‐h light/dark cycle).

ITGB3flox/flox (FLOX) mice were generated using clustered regularly interspaced short palindromic repeats (CRISPR)/Cas9 technology on a C57BL/6J background. ITGB3flox/flox mice were crossed with Albumin‐Cre mice to create hepatocyte‐specific ITGB3 knockout mice (HKO). The mice were confirmed by PCR with primer pairs (Table S2, Supporting Information).

For adeno‐associated virus (AAV8) (OBiO Technology, Shanghai, China)‐mediated Itgb3 overexpression, mice were fed with a HFD for 7 weeks then received an intravenous injection of 2.5×1011 virus particles of AAV‐Itgb3 or AAV‐GFP through the tail vein. To achieve AAV8‐mediated hepatocyte‐specific Dhhc5 overexpression, HKO mice were injected with AAV‐Dhhc5 through the tail vein. As control groups, FLOX or HKO mice were injected with AAV‐GFP. For AAV8‐mediated hepatic‐specific Itgb3 knockdown, 8‐week‐old HKO mice were injected with AAV‐shItgb3 (Hanbio Biotechnology, Shanghai, China) via the tail vein. The target sequence used against mouse Itgb3 was as follows: 5′‐GCTCATCTGGAGCTACTCAT‐3′. The sequence of 5′‐TTCTCCGAACGTGTCACGT‐3′ was used as a control‐shRNA.

To investigate the effects of Cyclo‐RGDfk (Apeptide Co., Ltd., China) on lipid metabolism, C57BL/6J mice on a high‐fat diet for 4 weeks received daily intraperitoneal injections of Cyclo‐RGDfk (1 or 5 mg/kg in PBS) or placebo. RGDfk was a well‐characterized, commercially available ITGB3 antagonist, and citations were now added to the Experimental Section confirming its high affinity and specificity.^[^ ^20^, ^21^ ^]^ After 4 weeks, mice were sacrificed, and serum levels of triglycerides (TG), total cholesterol (TC), alanine transaminase (ALT), and aspartate transaminase (AST) were measured. Liver sections were prepared for histological analysis. For evaluating the therapeutic effect of RGDfk on MASH, C57BL/6J mice were fed a high‐fat diet for 16 weeks, with RGDfk (5 mg/kg/day) administration during the final 4 weeks.

### Hepatic Extracellular Matrix Extraction

Hepatic extracellular matrix (hECM) was isolated as previously described.^[^ ^43^ ^]^ Mouse livers were harvested, rinsed in PBS, and agitated overnight at room temperature in 1% Triton X‐100 containing 20 mM EDTA (dissolved in ddH_2_O). The resulting hepatic ECM (hECM) preparations were washed five times with PBS and sterilized in 10% penicillin‐streptomycin‐amphotericin B solution (Gibco). Prior to use in cell cultures, hECM fragments were rinsed in serum‐free medium and homogenized using gentleMACS M tubes (Miltenyi Biotec).

### Machine Learning Algorithms

Data were analyzed following our established protocol.^[^ ^44^ ^]^ Using stratified sampling, the dataset was randomly split into training and testing subsets at a 7:3 ratio; the training subset was used for model development, and the testing subset for validation. Three machine learning algorithms—random forest (RF), generalized linear model (GLM), and support vector machine (SVM)—were implemented using the caret package (v7.1.0). Performance was evaluated using the DALEX package (v2.4.3), and residual plot analysis identified the RF algorithm as optimal for subsequent analyses.

### Glucose Tolerance Tests

Glucose tolerance was assessed in mice maintained on experimental diets for durations specified in relevant figures. For glucose tolerance testing (GTT), overnight‐fasted mice received intraperitoneal injections of glucose (1.5 g/kg body weight). Blood glucose levels were measured at 0, 15, 30, 60, and 120 min post‐injection using glucose test strips.

### Serum and Hepatic Biochemical Assays

Serum levels of TG, TC, ALT, and AST were determined using commercial kits (Nanjing Jiancheng Bio‐engineering, China). Selected samples were sent to a hospital laboratory for re‐testing to confirm result accuracy. To quantify hepatic TG and total cholesterol content, ≈50 mg of liver tissue was soaked in isopropanol and shaken overnight at 4 °C. Samples were then centrifuged at 12000 × g for 10 min, and the supernatant was used to measure total TG and TC using a commercial kit (Nanjing Jiancheng Bio‐engineering, China).

### Histological Analyses

Liver tissues were fixed in 10% neutral buffered formalin and paraffin‐embedded. Histological evaluation was performed on hematoxylin and eosin (H&E)‐stained sections. Frozen, OCT‐embedded liver sections were stained with Oil Red O (Wuhan Servicebio Technology, China) to quantify lipid droplet accumulation. Sirius Red staining (Wuhan Servicebio Technology, China) was used to assess collagen deposition and fibrosis severity.

### Cell Culture

AML‐12 and HepG2 cells were cultured in modified DMEM supplemented with 10% fetal bovine serum (FBS), 100 U/mL penicillin, and 100 U/mL streptomycin, and maintained at 37 °C in a 5% CO_2_ incubator.

To investigate the impact of extracellular matrix on hepatic lipid accumulation, AML‐12 cells were treated with isolated hECM for 24 h. Cellular TG content was quantified, and lipid droplets were visualized via BODIPY 493/503 staining.

### Quantitative Real‐Time PCR

Total RNA was isolated from samples using RNAiso Plus reagent (TAKARA, Japan). First‐strand cDNA was synthesized using 1 µg of total RNA as template with PrimeScript RT Master Mix (Vazyme, China). Quantitative real‐time PCR was performed using primers listed in Table S3 (Supporting Information), with β‐actin or GAPDH as endogenous controls. Gene expression levels were calculated using the 2^(‐ΔΔCt) method and presented as fold changes relative to the control group. Primer sequences for qPCR of specific genes are shown in Table S4 (Supporting Information).

### Western Blot

Tissue or cell samples were homogenized in RIPA lysis buffer (Beyotime, China) on ice. Protein concentrations were quantified using a BCA assay kit (Beyotime, China) according to the manufacturer's instructions. Equal amounts of protein lysates were separated by SDS‐PAGE (8–12% gels) and transferred onto polyvinylidene fluoride (PVDF) membranes. After blocking with 5% bovine serum albumin (BSA), membranes were incubated overnight at 4 °C with primary antibodies (diluted 1:500–1:1000 in TBST). Protein bands were detected using Immobilon Western Chemiluminescent HRP Substrate (Beyotime, China) and visualized with a Bio‐Rad imaging system.

### Immunoprecipitation (IP)

Tissues or cells were lysed in IP lysis buffer (Beyotime, China) and homogenized. The indicated antibody (1:100 dilution) was incubated with 30 µL of Protein A/G magnetic beads (MCE, China) overnight at 4 °C. Homogenates were then incubated with the antibody‐bound beads at room temperature for 1 h. Co‐immunoprecipitated proteins were collected, separated by SDS‐PAGE, and analyzed by mass spectrometry or Western blot.

### Immunofluorescence

Paraffin‐embedded human and mouse liver sections were used for immunofluorescence analysis. Sections were incubated overnight with primary antibodies, followed by a 1‐h incubation with fluorescent dye‐conjugated secondary antibodies. Sections were stained with DAPI and mounted with antifade solution. Images were acquired using a fluorescence microscope (Olympus).

### Metabolic Studies

Mice were singly housed in metabolic cages (Columbus Instruments) with ad libitum access to food and water. After a 24‐h acclimation period in respiratory chambers, mice were monitored in real time for 72 h to record parameters including food intake, water consumption, oxygen uptake, carbon dioxide emission, respiratory exchange ratio (RER), and energy expenditure, as previously described.^[^ ^45^ ^]^ All data were collected and analyzed using Oxymax software.

### Lipidomics Analysis

Lipidomic analysis was performed as previously described.^[^ ^46^ ^]^ For fatty acid quantification, mouse liver samples were analyzed using an Agilent 5977 quadrupole mass spectrometer (Agilent Technologies, USA) equipped with an electron ionization (EI) source and a MassHunter workstation. The optimized mass spectrometry conditions were as follows: injector temperature was 260 °C, quadrupole temperature was 150 °C, and the acquisition mode was single ion monitoring (SIM). The mass scanning range was m/z 30–550. Agilent gas chromatography system (Agilent 7820, Agilent Technologies, USA).According to the properties of the compounds, CP‐Sil 88 (100m x0.25mm x0.25 µm, Agilent, USA) gas chromatographic column was used, the injection volume was 1 pL, the split ratio was 10:1, the carrier gas was high purity helium, and the flow rate was 1.0 mL min^−1^. The initial temperature of the column oven was 100 °C for 5 min, and the temperature was programmed to 240 °C at 4 °C min^−1^ for 15 min. Data were acquired on the MassHunter GC/MS Acquisition (Agilent Technologies), and processed using Quant‐My‐Way (Agilent Technologies). Technical support for the analysis was provide by Sanshu Biotech Co., LTD.

### Determination of Fatty Acid Uptake

To assess in vivo fatty acid uptake, mice were injected intraperitoneally with BODIPY FL C16 (Invitrogen, D3821) at 0.5 mg/g body weight. Liver samples were collected 30 min post‐injection, homogenized in RIPA buffer, and centrifuged to obtain supernatants for subsequent experiments. Fluorescence intensity (FI) was measured immediately using a fluorescence microplate reader in black 96‐well flat‐bottom plates. Background signals were subtracted using readings from control‐treated mice, and data were normalized to the weight of the extracted tissue. Separately, mouse liver samples were dissociated into single cells and analyzed by flow cytometry.

For in vitro fatty acid uptake assays, BODIPY FL C16 uptake experiments were performed as previously described with modifications.^[^ ^47^ ^]^ HepG2 cells were cultured for 24 h in medium containing palmitic acid (PA, 200µmol/L) or BSA. Cells were incubated with BODIPY FL C16 at 37 °C for 30 min, washed three times with cold PBS, harvested using trypsin‐EDTA, and centrifuged at 600g for 3 min at room temperature. Samples were analyzed on a Beckman flow cytometer (Beckman Coulter) using FlowJo software (v10).

### Isolation of Plasma Membrane Protein

Plasma membranes from liver tissues or cells were isolated using a Membrane and Cytosol Protein Extraction Kit (Beyotime, China) according to the manufacturer's instructions. Briefly, tissues or cells were homogenized in lysis buffer containing protease inhibitors. To achieve complete lysis, samples were thoroughly homogenized using an ultrasonic device. Homogenates were centrifuged at 700g for 10 min to remove nuclear pellets. The supernatant was then centrifuged at 16 000g for 30 min at 4 °C to isolate total membrane protein fractions (including plasma membranes and organelles). The supernatant was discarded, and the residual pellet contained membrane proteins. Solution buffer was added, and samples were incubated at 4 °C for 30 min to ensure complete dissolution of membrane proteins. Samples were then centrifuged at 16,000g for 5 min at 4 °C, and the resulting supernatant contained membrane proteins. Loading buffer was added, and the mixture was incubated at 37 °C for 1 h for subsequent Western blot analysis.

### Protein Palmitoylation Analysis

Protein palmitoylation was assessed using the IP‐ABE method.^[^ ^48^ ^]^ Briefly, liver tissues and cells were lysed in RIPA buffer supplemented with protease inhibitors and 50 mM N‐ethylmaleimide. Lysates were immunoprecipitated using a CD36 antibody (Novus, NB600‐1423; 1:500 dilution) and Protein A/G Magnetic Beads (MCE, China). Each sample was split into two equal aliquots: one treated with hydroxylamine (HAM+) and the other untreated (HAM−). All samples were rotated at room temperature for 55 min to cleave thioester bonds in palmitoylated cysteines. Biotin‐BMCC was added, and samples were rotated at room temperature for an additional 55 min to specifically label palmitoylated cysteines. Subsequent immunoblotting entailed incubating blots with horseradish peroxidase‐conjugated streptavidin (1:10000 dilution) for 1 h at room temperature, with detection via chemiluminescence.

### Statistical Analyses

All data were processed using unpaired two‐tailed Student's t tests or one‐way ANOVA. Statistical significance was defined as p < 0.05. Analyses were conducted in GraphPad Prism v11 (GraphPad Software, San Diego, CA, USA).

## Conflict of Interest

The authors declare no conflict of interest.

## Author Contributions

Y.Z. and L.D. contributed equally to this work. Y.Z. designed and conducted the ex vivo analyses. Y.Z. and L.D. assisted in animal experiments. Y.Z., Z.C. and M.W. provided reagents and conceptual advice. W.D. provided the human samples. W.D. and H.J. contributed to data interpretation and provided technical support. Y.Z., L.D. and X.Z. critically reviewed the manuscript. Z.Y., W.D. and T.M. contributed to the discussion of results and critically reviewed the manuscript. X.Z.R., H.Z. and H.W. conceptually designed the study and supervised the project. X.Z.R., H.Z. and H.W. prepared and revised the manuscript.

## Supporting information



Supporting Information

Supporting Information

Supporting Information

Supporting Information

Supporting Information

## Data Availability

The data that support the findings of this study are available in the supplementary material of this article.
