# Supplementary figures and images for: Integrin β3 Orchestrates Hepatic Steatosis via a Novel CD36‐Dependent Lipid Uptake Complex
Source: Adv Sci (Weinh). 2025 Dec 8;13(8):e17455. doi: 10.1002/advs.202517455 (PMC12884801; doi:10.1002/advs.202517455)

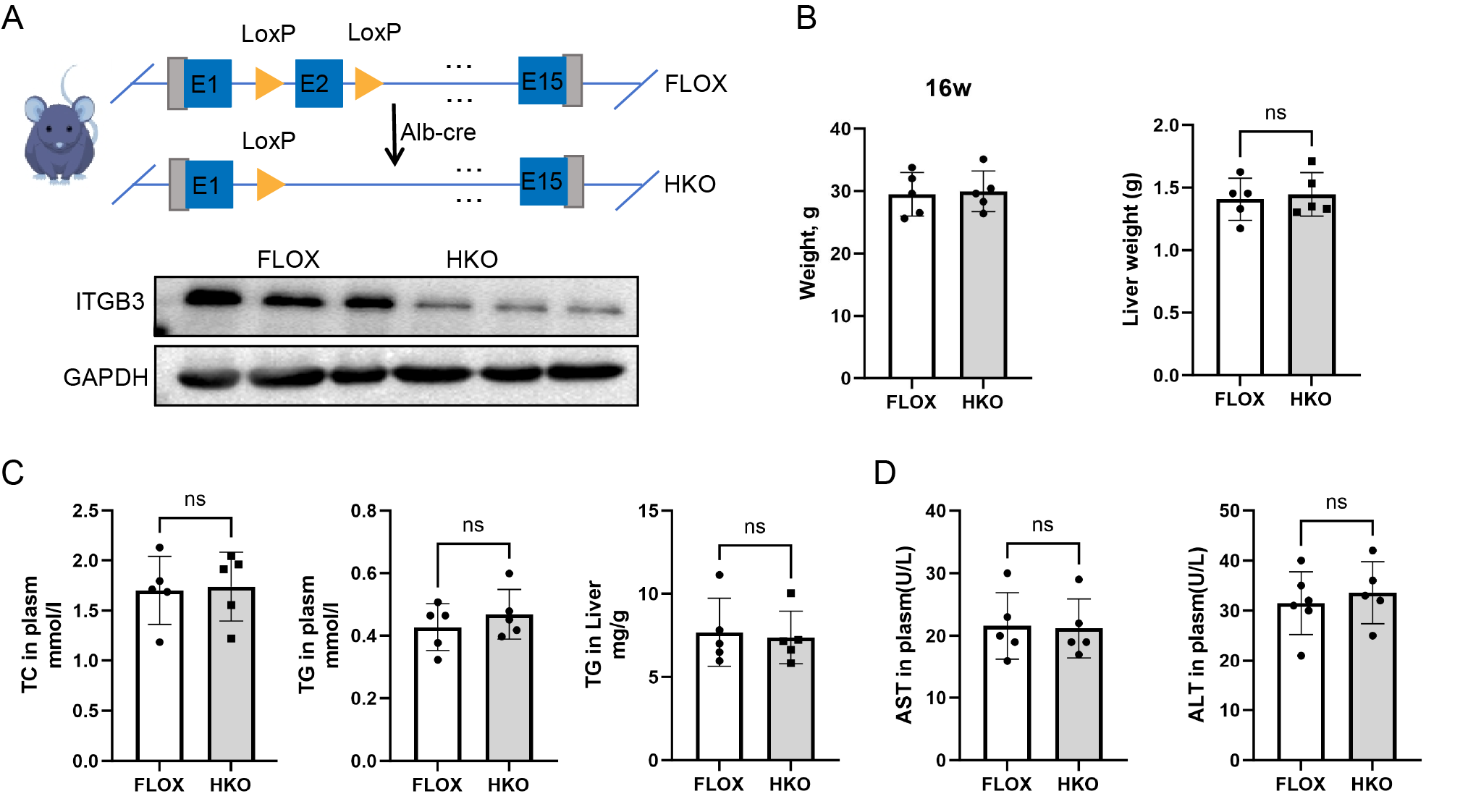

Supplement: Supplementary file 1 — Supporting Information [file ADVS-13-e17455-s002.png]

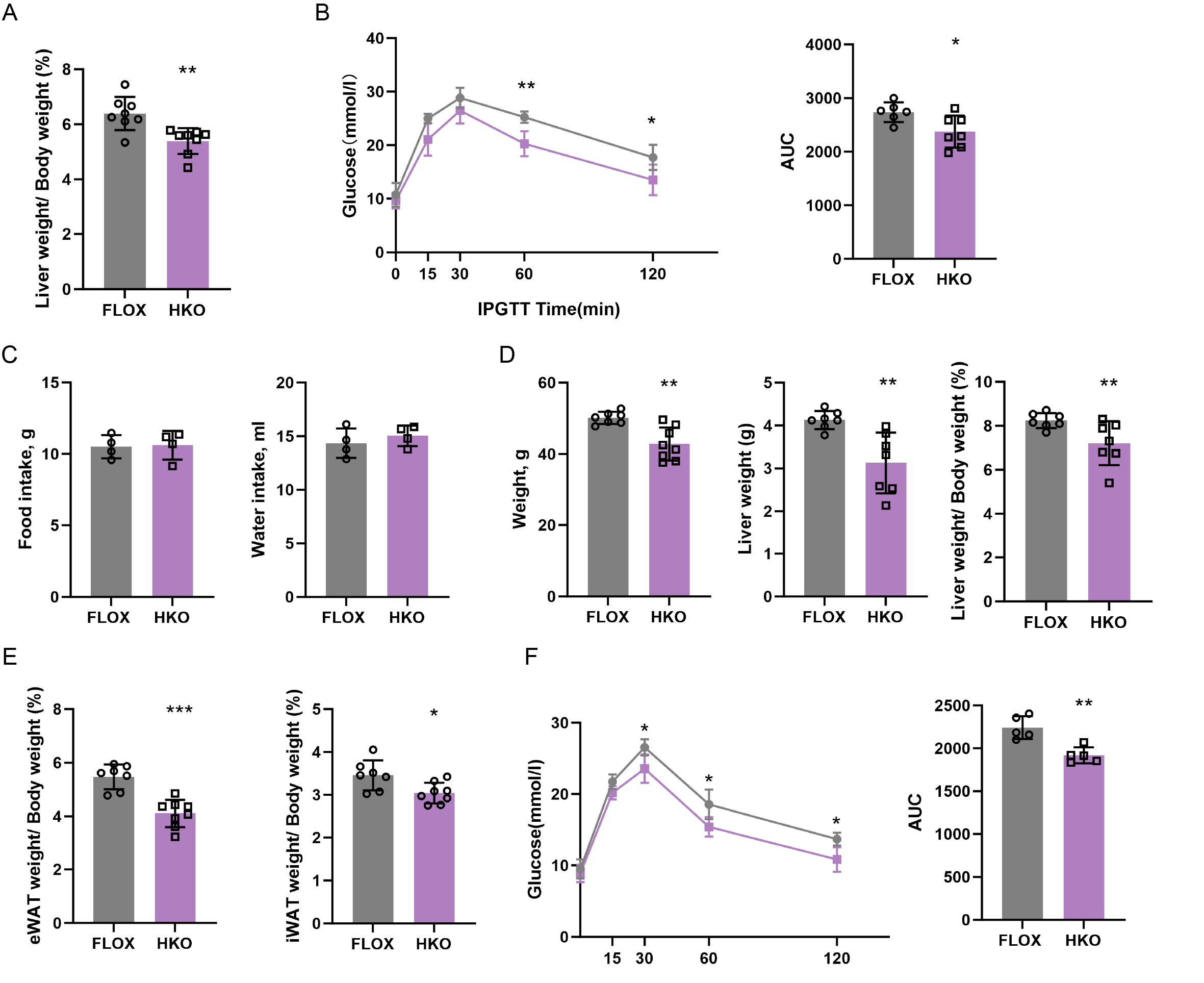

Supplement: Supplementary file 2 — Supporting Information [file ADVS-13-e17455-s005.png]

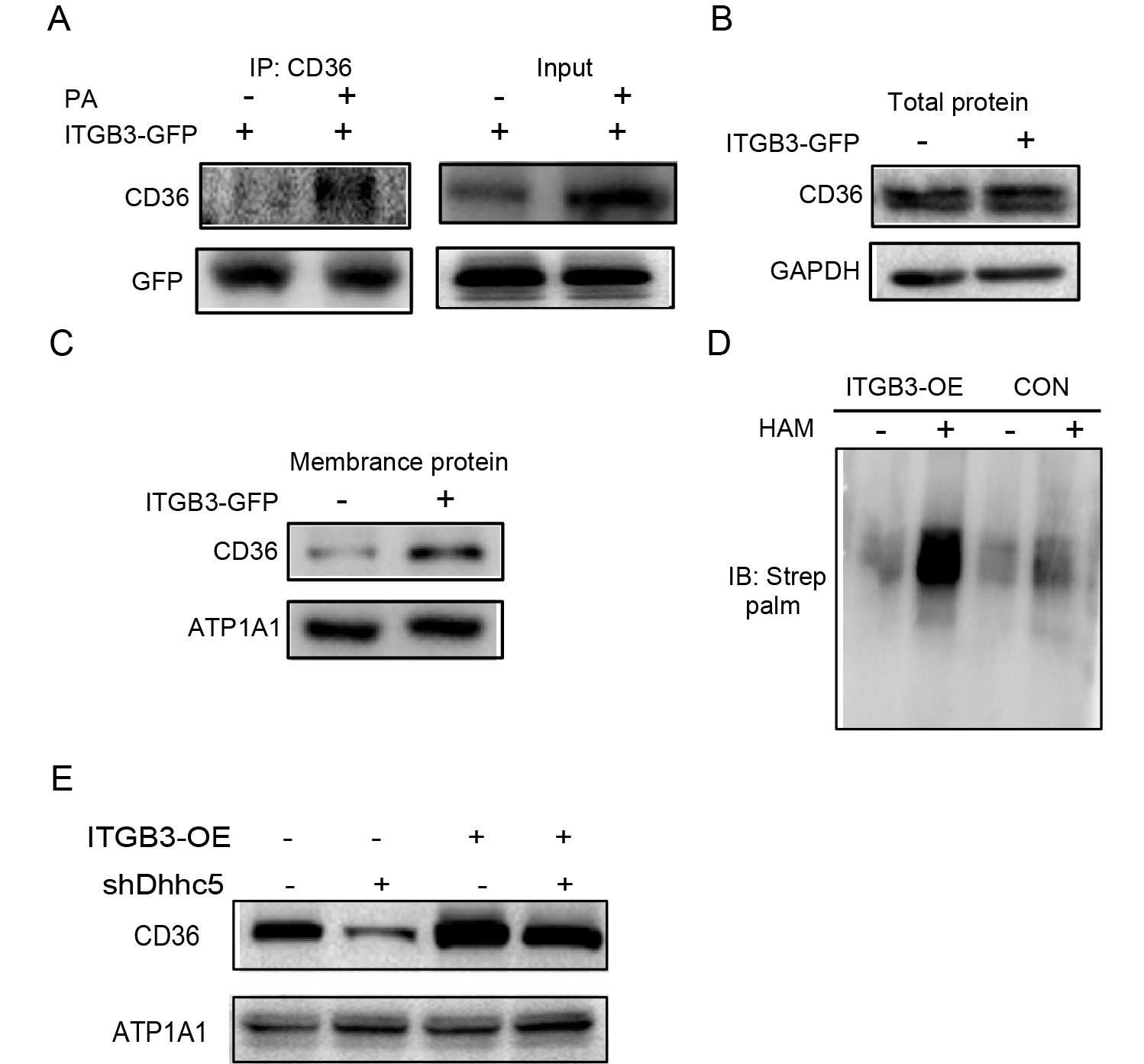

Supplement: Supplementary file 3 — Supporting Information [file ADVS-13-e17455-s004.png]

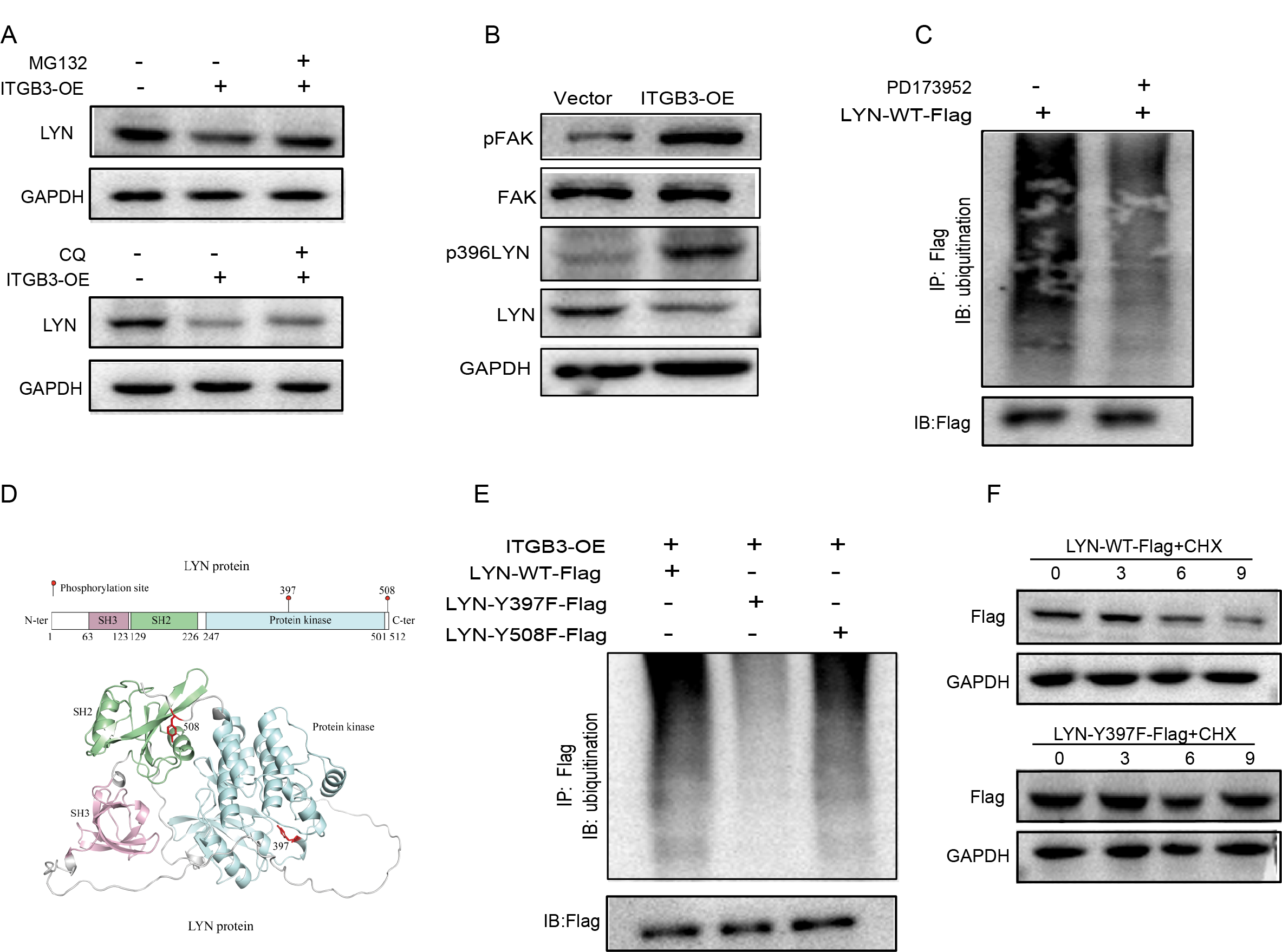

Supplement: Supplementary file 4 — Supporting Information [file ADVS-13-e17455-s001.png]

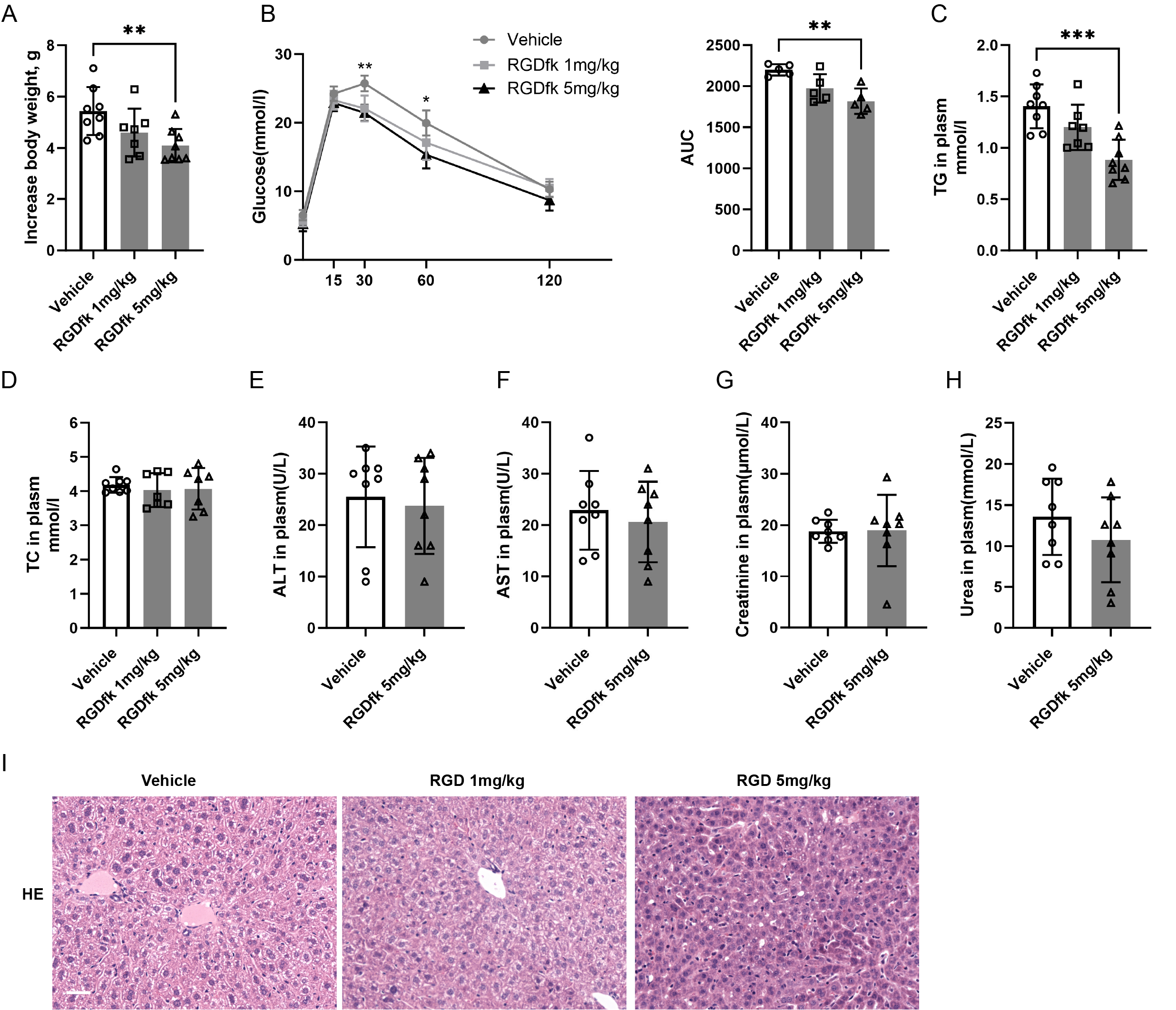

Supplement: Supplementary file 5 — Supporting Information [file ADVS-13-e17455-s003.png]
